# Humanized Mice Are Precious Tools for Preclinical Evaluation of CAR T and CAR NK Cell Therapies

**DOI:** 10.3390/cancers12071915

**Published:** 2020-07-15

**Authors:** Rana Mhaidly, Els Verhoeyen

**Affiliations:** 1 C3M, Université Côte d’Azur, INSERM, 06204 Nice, France; rana.mhaidly27@gmail.com; 2 Institut Curie, Stress and Cancer Laboratory, Equipe Labellisée par la Ligue Nationale Contre le Cancer, PSL Research University, 26, Rue d’ULM, F-75248 Paris, France; 3 Inserm, U830, 26, Rue d’ULM, F-75005 Paris, France; 4 CIRI, Université de Lyon, INSERM U1111, ENS de Lyon, Université Lyon1, CNRS, UMR 5308, 69007 Lyon, France

**Keywords:** CAR T cell, CAR NK cell, PDX mouse, humanized mouse model, xenograft mouse, cancer therapy, in vivo gene therapy

## Abstract

Chimeric antigen receptor (CAR) T-cell therapy represents a revolutionary treatment for hematological malignancies. However, improvements in CAR T-cell therapies are urgently needed since CAR T cell application is associated with toxicities, exhaustion, immune suppression, lack of long-term persistence, and low CAR T-cell tumor infiltration. Major efforts to overcome these hurdles are currently on the way. Incrementally improved xenograft mouse models, supporting the engraftment and development of a human hemato-lymphoid system and tumor tissue, represent an important fundamental and preclinical research tool. We will focus here on several CAR T and CAR NK therapies that have benefited from evaluation in humanized mice. These models are of great value for the cancer therapy field as they provide a more reliable understanding of sometimes complicated therapeutic interventions. Additionally, they are considered the gold standard with regard to assessment of new CAR technologies in vivo for safety, efficacy, immune response, design, combination therapies, exhaustion, persistence, and mechanism of action prior to starting a clinical trial. They help to expedite the critical translation from proof-of-concept to clinical CAR T-cell application. In this review, we discuss innovative developments in the CAR T-cell therapy field that benefited from evaluation in humanized mice, illustrated by multiple examples.

## 1. Introduction

### 1.1. Anti-Cancer CAR T Cell Therapy

**Figure 1:**
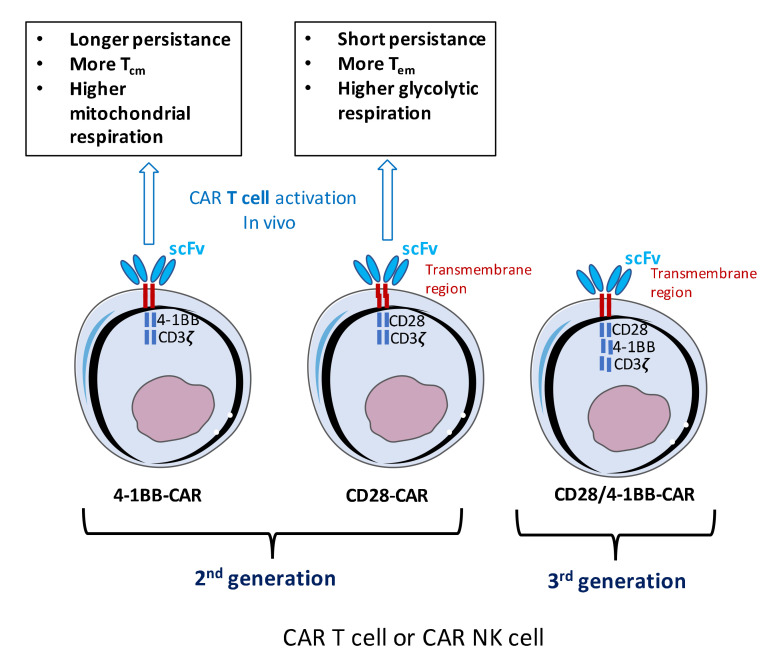
Chimeric antigen receptor (CAR) T cell engineering using different CAR designs and their in vivo persistence. Second-generation CAR T cells containing a CD3 zeta signaling domain, a CD28 or 4-1BB co-stimulatory domain and a scFv that will be displayed at the surface of the T cell for anti-cancer antigen recognition. For the second-generation CARs is indicated their dependence on a metabolic pathway and their persistence in vivo according to the co-stimulatory domain used. The third generation CAR contains 2 co-stimulatory domains.

Despite progress made in the treatment of many leukemias, lymphomas, and solid cancers, therapeutic outcomes remain refractory and better treatment options are required. A recent successful anti-cancer strategy is based on engineered T cells called chimeric antigen receptor T-cell (CAR-T) therapy [1]. CAR T-cell therapy involves changing a patient’s own immune cells to augment the immune response to cancer cells [2]. CARs are synthetic proteins consisting of a specific antibody binding domain, usually a single-chain variable fragment (scFv) recognizing a cancer antigen that is combined with the effector function of T cells (Figure 1). First-generation CARs carried one cytoplasmic signaling domain (e.g., the Fc receptor G chain or CD3ζ). These did not demonstrate robust anti-tumor effects and became anergic [3,4,5]. Optimized CAR T design resulted in second- and third generation CARs, in which additional costimulatory domains were inserted such as CD28, 4-1BB, ICOS, and OX40 alone or in combination [6,7] (Figure 1). This CAR design mimicked natural TCR co-stimulation and enhanced CAR T cell function [8]. CAR T cells contain for example an extracellular scFv, linked by a transmembrane domain to CD28 and/or 4-1BB co-activation domains and the CD3ζ intracellular signaling domain [9] (Figure 1).

However, the choice of the co-stimulatory domain has important consequences. In some clinical trials for B-CLL, CAR T carrying the CD28 or 4-1BBζ costimulatory domains had very different outcomes. The latter domain allowed long-term persistence of CAR T cells (sometimes for years) and avoided exhaustion of the CAR T cells within some patients, while CD28 allowed CAR T cell to survive only for 30 days in the patients [10,11,12,13]. A possible explanation was provided by the fact that 4-1BB CAR T cells showed enhanced survival and higher frequency of central memory T cells, which relied on mitochondrial respiration for their energy requirements [14]. In contrast, CD28 CAR T cells induced more effector memory T cells relying on the activation of the glycolytic pathway to provide energy for their proliferation and function (Figure 1). This underlines the importance of choice of the co-stimulatory domain(s). In accordance with this notion, in cases with a subsequent complete response, the infused CD8^+^ CAR T cells depended more on mitochondrial respiration as compared with non-responders, which positively correlated with the expansion and persistence of CAR T cells [15].

Ongoing clinical trials have described durable rejection of previously refractory B-cell malignancies including chronic lymphocyte leukemia (CLL [16,17]; 51–77% remission), acute lymphocyte leukemia (ALL [13,18]; 68–93% remission) and diffuse large B cell lymphoma (DLBCL [19,20]; 68–86% remission), in patients after CD19-directed CAR therapy [10,21,22,23]. A complete response rate as high as 93% was obtained in leukemia patients. In 2017, this has led to the acceptance of two CAR T cell therapies by the regulatory agencies in the USA (Food and Drug agency; FDA) and Europe (European Medicine Agency; EMA) for B-cell leukemia. Along with CD19 CAR-T cells, other CAR-T cells directed against CD5, CD33, CD70, CD123, CD38, and B cell maturation antigen (BCMA) are under evaluation for hematological malignancies (HM) [24,25]. In this regard, CD5 presents a potential target in T-ALL and malignancies involving the subpopulation of B cells called B1 cells [24,26,27]. CD33 is a target in myeloid malignancies, especially acute myeloid leukemia (AML), and CD123 is expressed in different HM, including blastic plasmacytoid dendritic cell neoplasm, hairy cell leukemia, B-ALL, and AML [24,28]. CD38 and BCMA are mostly expressed on myeloma cells. The successful application of CARs directed against hematological malignancies has more recently encouraged the application to other cancers including solid cancers [25]. CD70 for example has a broad spectrum of expression in HMs and solid tumors [29,30]. CAR T cells directed against more than 20 different biomarkers are currently being evaluated in clinical trials including CAR T cells to treat solid tumors [24]. The successful experience with CAR-expressing T cells in the treatment of hematological malignancies has revolutionized the field of immunotherapy. T cells modified for chimeric antigen receptor (CAR) expression that recognizes a specific antigen on the surface of malignant B cells, such as CD19, is one of the biggest steps forward in conquering cancer [16,19,31]. It is important though to mention that severe side effects emerged in CAR T cell trials, such as cytokine release syndrome (CRS) or graft-versus-host disease (GvHD) [22,32]. Importantly, CRS in CAR T cell treated patients is nowadays better controlled and is managed according to the grade of CRS severity [33]. Severe CRS is managed for example by administration of anti-inflammatory molecules such anti-IL-6 receptor (tocilizumab) and anti-IL6 antibodies (siltuximab) among other various therapeutic interventions [33,34,35,36,37].

### 1.2. Anti-Cancer CAR NK versus CAR T Cell Therapy

More recently, interest grew to develop similar approaches for other immune cell subsets, such as natural killer (NK) cells. Allogenic NK cells are an attractive option for CAR expression because they have cytotoxic functions and spontaneously demonstrate anti-cancer effects [38,39,40]. Moreover, infusion of allogenic NKs into patients proved to be a safe immunotherapy in cancer patients [41,42]. Contrary to T cells, natural killer (NK) cells kill their targets in a non-antigen-specific manner and do not carry the risk of inducing GvHD [39]. Hence, unlike CAR T cells that require autologous T cells, therapeutic CD19-CAR-NK cells could be generated as an off-the-shelf product from healthy donors and hold the potential of also attacking CD19-negative leukemia cells through natural cytotoxicity mechanisms [43].

NK cells express multiple cytotoxicity receptors, for which the ligands are overexpressed on tumor cells and cells from the tumor microenvironment (TME). NK cells are thus good candidates to reduce specifically the number of tumor suppressor cells in the TME and reactivate a strong anti-tumor response. One particular problem observed is that often these ligands for NK cytotoxic receptors are downregulated in the TME. For this reason, arming the NK cells with a CAR could improve their function. Compared to CAR T cells, CAR NK cells will target tumor cells via multiple mechanisms with less pro-inflammatory cytokine release and thus less risk of inducing a cytokine storm. Another important point is the fact that CAR-NK cells are short-lived [44,45], which might represent an advantage when targeting T cell malignancies such as peripheral T cell lymphomas to avoid a persistent immuno-suppression in the patients (see below Section 3.3). Several preclinical studies have directed CAR NK cells against tumor targets such as CD19 [46], CD20 [47], CD244 [48] and HER2 [49].

However, despite many advantages of NK cells as a cellular therapy, one of the major obstacles to use NK cells in immunotherapy is the lack of an efficient gene transfer method for primary human NK cells. Viral gene delivery to primary NK cells has always proven very challenging reaching at best 10%. Very recently, though, two independent studies have shown that this hurdle can be overcome by changing the vesicular stomatitis G (VSV-G) envelope glycoproteins (gps) at the surface of a lentiviral vector (LV) by a baboon retroviral envelope (BaEV-LV [50,51]). These new surface-modified BaEV-LVs allowed with ease up to 80 % gene modification of activated NK cells and even up to 30 % of freshly isolated NKs [52,53]. BaEV-LVs were shown to generate functional CAR expressing NKs. Another study also showed high-level CAR delivery into NK cells employing an alpha-retroviral vector system [54]. These results will pave the way to move CAR NK cell therapy into the clinic. Especially, since a first clinical trial using CD19-targeted CAR NK cells resulted in a high response rate and an excellent safety profile [55]. Nevertheless, CAR NK cell therapies, though they seem promising anti-cancer drugs, have not yet been accepted by the regulatory agencies in the USA and Europe for clinical use at this moment.

## 2. Different Humanized Mice Models for Preclinical Testing of CAR T and NK Cell Therapy

To fully understand CAR therapy in terms of its limitations and capacities, preclinical testing and in vivo evaluation in a humanized mouse model has become a gold standard to validate these cell therapies and get regulatory approval. One of the most widely used models is the immunodeficient non-obese (NOD)/SCIDγc^−/−^ (NSG) mouse, which supports development of a human hematopoietic and immune system [56]. Since NSG mice are devoid of murine T, B, NK, and functional DCs, they easily accept engraftment of human cell lines, healthy and tumor tissues. This mouse model has become the platform to study the interaction between the human blood system and cancer cells.

### 2.1. Xenograft Mouse Model for CAR T and CAR NK Cell Evaluation

**Figure 2:**
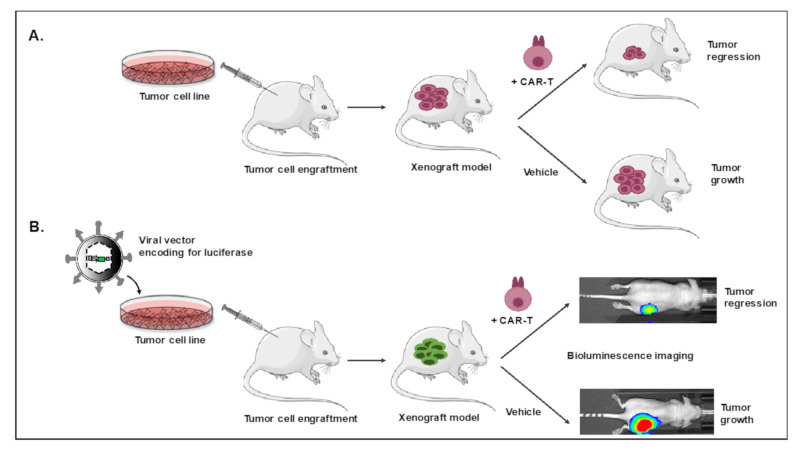
Tumor cell xenografted humanized mice for CAR T cell evaluation. Tumor cell lines not transduced (**A**) or transduced (**B**) with a vector encoding for the reporter gene, luciferase, are injected intravenously or subcutaneously into NOD/SCIDγC^−/−^ (NSG) mice. After tumor development, T cells modified with a CAR against a specific antigen on the tumor cells are injected. A follow-up of tumor size via bioluminescence or measurement of tumor size is performed to evaluate CAR T cell efficacy and mice survival is evaluated. At endpoint, CAR T cell infiltration, cytokine release and T cell immune phenotypes (exhaustion markers, persistent T cell markers) are determined.

Two major humanized mouse models are used for evaluation of CAR T cells. The first one is the xenograft mouse model, in which a human tumor cell line is engrafted in the immune-compromised mice (mostly the NSG model), followed by infusion of human CAR T cells (Figure 2A). To facilitate in vivo follow-up, NSG mice are often injected intra-dermally or intravenously with a luciferase-expressing tumor cell line providing easy measurement of tumor growth by bioluminescence imaging [57,58] (Figure 2B).

Using a xenograft mouse model, anti-CD19 directed CAR T cells were shown to eliminate the CD19^+^ cancerous B cells, resulting in prolonged survival of this NSG leukemia xenograft model [59] and this set the basis for the first clinical trial and later on, approval of the first CAR T cell products by the FDA.

It is important to emphasize that the human cancer cell line xenograft models lack a functional human immune system and other human tissues, which may modulate the anti-cancer activity in vivo. In addition, they are not representative of heterogeneous tumors. Moreover, valid safety studies require that the expression profile the murine tumor antigen is identical to that seen in humans, which is not always the case. Nevertheless, xenograft models were instrumental to establish a first proof of concept. Multiple studies use xenografted mice for CAR T cell evaluation and we will report hereafter more specific applications (see Section 3).

### 2.2. Patient-Derived Xenograft Model for CAR T/NK Cell Evaluation

**Figure 3:**
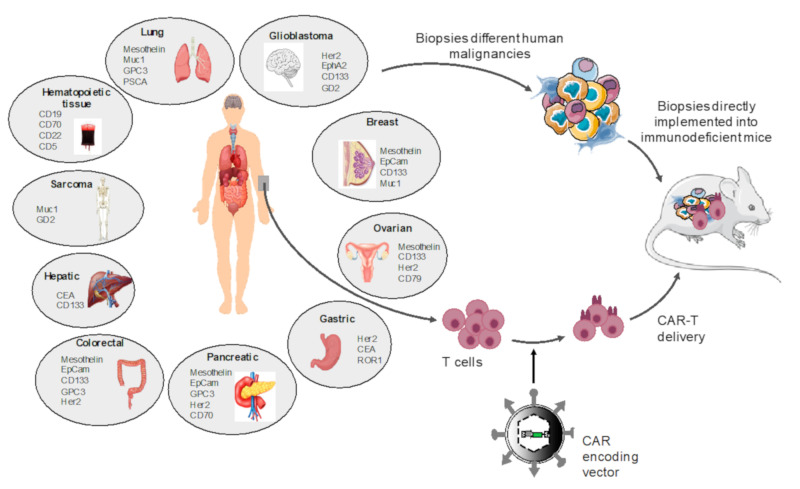
Patient-derived-xenograft mice for CAR T cell evaluation. Patient tumor biopsies are injected into NSG mice and subsequently T cells from the same patient are modified using a vector encoding for a CAR directed against a specific antigen present on the tumor cells and infused in the patient-derived xenograft. A follow-up of tumor size or measurement of subcutaneous tumors is performed to evaluate CAR T cell efficacy and mice survival. At endpoint CAR T cell infiltrations, cytokine release and T cell immune phenotypes (exhaustion markers, persistent T cell markers) are determined. Tumor-associated antigens (TAA) for each specific tumor type are indicated. Note that not all CAR T cells directed against these TAAs were evaluated in PDX models.

Another more pertinent humanized mouse model was established for evaluation of CAR T cell efficacy. This second model is called a patient-derived xenograft (PDX) model and consists of injection of a primary tumor biopsy from a patient instead of human cell lines and infusion of matched patient CAR T cells (Figure 3). In the PDX model, the tumor cells as well as the tumor microenvironment (e.g., immune cells) are present in vivo. Therefore, these models are increasingly used for evaluation of personalized anti-cancer T cell therapies and are more relevant than cancer cell line xenograft models for translation to the clinic.

About 10 years ago, a matched patient’s tumor and T cells were engrafted in NSG mice to show the cancer patient’s T cells can function as CAR T cells [60]. Another study showed the efficacy of CAR T cells in PDX mice for 3 different patient hepatocarcinoma tumors. Two PDX mice eradicated the tumors upon CAR T cell therapy, while one was resistant and showed upregulation of checkpoint inhibitor molecules [61]. This can predict that a combinatorial CAR T and check point inhibitor treatment might be recommended in this particular patient. PDX models allow indeed to asses to some extend human immune responses to primary cancer cells, which is highly relevant for clinical translation. Nevertheless, the transfer of human mature T cells into NSG mice such as performed in the PDX model, usually leads to GvHD, which does not allow long-term follow-up of CAR T cell therapy efficacy [62,63]. In contrast, Haworth et al. [64], showed that NSG mice reconstituted with human CD34^+^ stem and progenitor cells gave rise to in vivo murine-matured human CD3^+^ T cells, which can be isolated, genetically modified and reinfused into the same mice. No GvHD was detected in these mice and they might therefore represent a better model for longterm evaluation of CAR T cell-based treatments in preclinical settings in the future.

### 2.3. Fully Autologous Humanized Cancer Model for CAR T Cell Testing

Still these PDX models do not possess a fully functional human immune system and they cannot fully predict what might happen in cancer patients. Very recently a more complex humanized mouse model was developed. Jin et al. developed a mouse model for human B-ALL, in which cancer and immune cells are autologous [65]. Firstly, they engrafted in one NSG recipient, hCD34^+^ human progenitor cells and a human fetal thymus to generate a humanized mouse with human immune-competence since T-cell are educated on the co-transplanted human thymic tissue. In parallel, a second mouse was engrafted with fetal CD34^+^ cells from the same human donor transduced with a B-ALL relevant oncogene and developed human B-ALL. Then they engrafted these autologous B-ALL cells into the firstly developed immune-competent humanized mice to have a valid B-ALL model mimicking closely the patient situation. In a next step, they produced matching human anti-CD19-CAR T, which were then reinfused into the B-ALL human like mouse model.

This model has important unique characteristics: (1) a human functional immune system; (2) autologous B-ALL tumor cells, (3) the CAR T cells are modified autologous T cells and they are educated on a human thymus. All these characteristics together resulted in a mouse model that, though complex in its generation, was highly adapted to evaluate human CAR T cell efficacy, resistance and toxicity.

However, from a practical point of view it will not be evident or feasible to establish such an autologous model for many different cancer types.

## 3. Preclinical Evaluation of CAR T and CAR NK Cell Therapies in Humanized Mice

**Figure 4:**
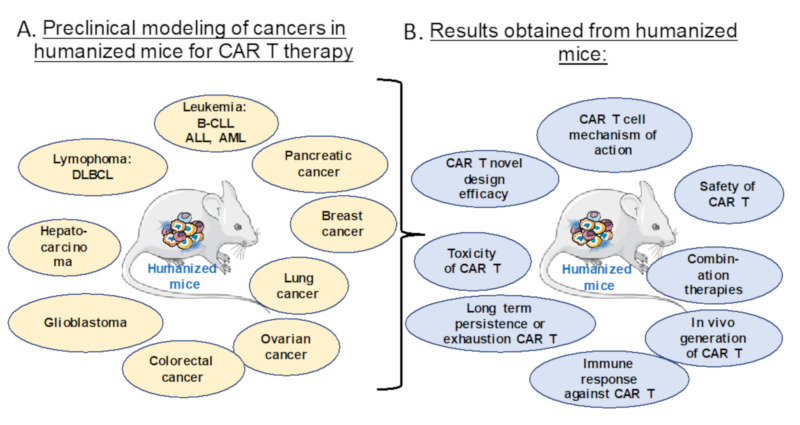
Preclinical modeling of CAR T Cell therapy in humanized cancer mice models. (**A**) Different humanized mice models for preclinical modeling of different malignancies. (**B**) The relevant preclinical data for CAR T cell treatment obtained for the indications in (**A**).

Multiple cancer cell lines, patient malignant blood cells or solid cancer biopsies can be transplanted into the NSG mice model (Figure 4A) for preclinical evaluation of CAR T cell therapies in terms of efficacy, safety, persistence, exhaustion, toxicity and immune response, which is illustrated here by multiple examples (Figure 4B).

### 3.1. Evaluation of Safety and Toxicity of CAR T or NK Cells in Humanized Mice

Safety of CAR T/NK cell therapy is an important issue. Especially since during the ex vivo transduction and expansion an unwanted transfer of the CD19-CAR into one single leukemic cell has led to relapse and death of a patient with a B cell malignancy [66]. Moreover, a serious toxic side-effect of CAR T cell therapy is cytokine release syndrome and/or off-tumor/on target toxicity as already reported [34].

Liu et al. [67] therefore build a safety measure into their CAR NK cells. Interestingly, they used cord blood NK cells which they equipped with an anti-CD19 CAR, an IL-15 expression cassette and an inducible caspase-9 suicide gene [68] to be able to eliminate the CAR NK cells in case of an adverse event. IL-15 helps to conserve the stem cell memory T cell phenotype. Notably, IL-15 released from these CAR NK cells significantly improved their anti-tumor function, proliferation and persistence in a Raji B cell lymphoma xenograft model [67]. In the same mouse model, these authors demonstrated that in case of CAR NK toxicity, they were able to activate the suicide gene by injecting a small dimerizer molecule, which induced rapid and efficient elimination of the CAR NK cells in vivo [67]. These anti-CD19 CAR NK cells derived from cord blood with a build-in safety switch are currently evaluated in clinical trials (NCT03690310).

GD2 is a ganglioside antigen expressed on the surface of several solid cancer such as neuroblastoma, glioma, cervical cancer and sarcoma [69,70]. Some clinical trials were extremely successful using GD2 directed CAR T cells for neuroblastoma showing even long-term persistence of CAR T cells [71,72]. However, GD2 is also expressed on healthy neurons, melanocytes and nerve fibers [69,73]. Thus, there is some concern that central nervous system toxicity might be caused due to CAR T cell mediated neural destruction. Richman et al. [74], in an attempt to increase anti-GD2 CAR T cell efficacy introduced into the anti-GD2 scFv a single point mutation. This new GD2-CAR design showed an enhanced anti-tumor activity against human neuroblastoma xenografts in NSG mice. However, those mice with the higher tumor reduction, experienced severe brain toxicity [74]. Strong infiltration of CAR T cells was found in the brain of this humanized cancer mouse model associated with destruction of neurons. These results give a serious warning that cancer antigens expressed on essential normal healthy cells is problematic and needs careful attention. Modifications, even minor, in CAR design, might raise safety problems. Although other anti-GD2 CAR designs [75] did not report brain toxicity in again other xenograft models of neuroblastoma, caution is warranted and a careful preclinical evaluation might reveal toxicities before entering into a GD2-CAR T clinical trial. Needless to emphasize that Phase I CAR T trials targeting neuroblastoma are focusing on safety of the treatment (NCT02107963). As mentioned above for anti-CD19 CAR NK cells, it might be advisable to introduce a suicide gene to eliminate the GD2-CAR T in case of severe toxic side-effects.

### 3.2. Efficacy of Novel ‘Optimized’ CAR Designs in Humanized Mice

For the moment, it is still not completely clear why some CAR T cells persist or not in patients? Phenotypic changes, exhaustion, poor tumor targeting, immunity, off target toxicity in vivo might all influence the outcome of CAR T cell therapy. To be able to rationalize new CAR T cell designs and their production, a method for tracking these cells in vivo would provide invaluable information on toxicity and pharmacodynamics in the treated patients. Moreover, this might speed up the translation of CAR T cell therapy in an allogenic setting.

With the objective to facilitate isolation and follow-up of CAR T cell persistence in vivo upon administration to the patients, Cassucci and colleagues [76], included an extracellular spacer within CAR itself based on the low-affinity nerve growth factor receptor (NGFR), lacking its intracellular signaling domains [77,78]. Firstly, this allowed to enrich the CAR^+^ T cells by a simple anti-NGFR magnetic bead selection. Secondly, NGFR-spaced CAR T cells directed against CD44 variant 6, allowed upon infusion into clinically relevant (THP-1 luciferase^+^ or MM.1S-luciferase^+^) xenografted NSG mice, tracking of these CAR T cells by flow cytometry analysis using an anti-NGFR antibody. This permitted in the NSG xenografts to follow how the CAR T cells expanded, persisted and induced a strong antitumor activity against leukemia and myeloma [76]. As a safety feature these authors also included a suicide gene (thymidine kinase) into the CAR construct, to eliminate the CAR T cell upon an adverse event for example in an allogenic setting when GvHD occurs [79]. Indeed, administration of the drug ganciclovir eliminated in the NSG xenografted mice efficiently the CAR T cells. Since CD44v6 is overexpressed in acute myeloid leukemia and multiple myeloma [80,81], the NGFR-spacer containing CD44v6 CAR T cell, equipped with a suicide gene entered recently into clinical trials for these indications (NCT04097301). Interestingly, Weist et al. [82] approached the same question by labeling CAR T cells with ^89^Zr-oxine [83,84] before infusion into two xenograft tumor models for: 1) glioblastoma, in which they administrated ^89^Zr-oxine labeled CAR T cells targeting the IL13Rα2 epitope present on this brain tumor and 2) a subcutaneous prostate tumor NSG model, in which they injected prostate stem cell antigen-targeted CAR T cells [82]. Imaging by positron emission tomography (PET) allowed with high sensitivity to track the CAR T cells in vivo for tumor tropism and distribution in a quantitative manner according to the administration route of the CAR T cells. Brown et al. [85], indeed showed that IL13Rα2 targeted CAR T cells improved anti-tumor efficacy against glioblastoma especially upon local intracranial delivery. Therefore, CAR T cell tracking might clearly allow to predict the effect of CAR T cell design and administration route on in vivo performance for clinical applications.

To minimize the risk of antigen escape by leukemic cells that lost CD19 expression, a bi-specific CAR was designed targeting two B-cell specific molecules, CD19 and CD20. In contrast to CD19-CAR T cells that only targeted CD19^+^ leukemic cells, the bispecific CD19/CD20-CAR T cells also eradicated all leukemic cells, even those that lost CD19 expression at the surface in a xenograft NSG model [86]. Interestingly, anti-CD19 CAR T cell therapy was evaluated in a B-CLL xenograft mice model by injecting into mice B cell lines carrying individual KOs representative for the mutational landscape in B-CLL [87]. In vivo, they confirmed that anti-CD19 CAR T cells prolonged survival of the different genetic classes of B-CLL tumor cells and revealed a differential anti-tumor efficacy according to the mutation introduced [87]. This emphasizes the need for more personalized and optimized CAR design in treatment of B-CLL.

### 3.3. Persistence and Exhaustion of CAR T and NK Cells in Vivo

CAR T cells are usually generated from PBMCs and expanded by culture in the presence of IL-2 [88]. However, this means that the autologous T cells after expansion are phenotypically heterogeneous and consist mostly of highly differentiated T cells: effector memory (T_em_) or effector T cells (T_eff_), which are prone to exhausting and do not readily persist in vivo. In contrast, when less differentiated naive or stem cell memory T cells (T_scm_) were engineered for CAR expression, these induce more potent antitumor responses than the previously mentioned T cell subsets [89,90,91]. IL-7 and IL-15 culture or expression of IL-15 by the CAR T cell itself, seemed to preserve T_scm_ cells expressing CARs [92,93]. Therefore, the objective of Alizadeh et al. [94] was to produce CAR T cells that are less exhausted and less differentiated by expanding them in the presence of IL-15. These authors confirmed the preservation of T_scm_ CAR T cells in the presence of IL-15 as compared to IL-2. Moreover, they expanded anti-CD19 CAR T cells in IL-15 and IL-2 and administrated them to NSG mice xenografted with a luciferase marked Raji B cell line. The IL-15 expanded CAR T cells outperformed by far the ones expanded in IL-2 in terms of antitumor potency and persistence in vivo in this model [94]. Interestingly, this can probably be contributed to the fact that IL-15 expanded CAR T cells downregulate their mTORC activity, leading to a metabolic switch in the CAR T cells from glycolysis to mitochondrial respiration, a hallmark of persisting memory T cells [14,15,94]. Additionally, this IL-15 effect was independent of the CAR design (costimulatory domain CD28 or 4-1BB) and CAR T cell target (anti-CD19 CAR for B cell targeting or anti-IL-13Ra2-CAR for glioblastoma). This opens the possibility to improve future CAR T cell therapies.

Heczay and coworkers [95], selected yet another T cell subset for CAR expression, the CD1d-restricted natural killer T (NKT) cells. These have intrinsic anti-tumor properties and CD1d is expressed on only a few cell types, limiting potential toxicity (GvHD) in autologous or allogenic settings [96]. These authors equipped the NKT cells with a CAR against GD2 ganglioside, highly expressed on neuroblastoma [71]. Especially the third generation GD2 CAR design with both the CD28 and 4-1BB costimulatory domains enhanced in vivo persistence of these NKT CAR cells and revealed potent antitumor activity in a xenograft NSG model of metastatic neuroblastoma, including a human blood system. This model closely mimics what is detected in the patients, since the hematopoietic system is required for growth/maintenance of NB tumors. Repeated administration of the NKT CAR cells, not possible with CAR T cells due to rejection, increased survival of this mouse model without inducing GvHD.

In some specific applications, one could prefer transient CAR expression over persistent expression in vivo. Peripheral T cell lymphomas (PTCLs), for which no effective treatment options exist and outcome is very poor, became very recently a target disease for CAR T cell therapy. A recent overview of the mechanisms involved in different PTCLs and the many novel drugs under evaluation underlines the difficulty to find effective targeted treatments [97,98]. Therefore, targeting of malignant CD4^+^ T cells in these T cell lymphomas by an anti-CD4 CAR T cell therapy is considered an option [99]. Engineered anti-CD4 CAR CD8^+^ T cells displayed a significant anti-leukemic effect in vivo in a xenograft NSG mouse engrafted with the KARPAS 299 aggressive PTCL cell line. However, since anti-CD4 CAR T cells can persist over months or years, patients might suffer from immunodeficiency due to a severe side-effect: the prolonged elimination not only of malignant but also healthy CD4^+^ T cells. In this particular application, one could prefer transient CAR expression over persistent expression in vivo. Since in contrast to CAR T cells, CAR NK cells have a limited lifespan, with a turn-over of about 2 weeks [44], they might be the target cell of choice for anti-CD4 CAR therapy in PTCL. It is expected that these CAR NK cells disappear shortly after eliminating the cancer cells and thus have a lower risk of long-term toxicity. Pinz et al. demonstrated that anti-CD4 CAR NK cells significantly prolonged survival of PTCL xeno-grafted mice by lysis of the tumor cells [99]. This opens a new avenue of curative treatments for PTCL patient with no or little therapeutic options. Since CD4 is expressed at high level on acute myeloid leukemia cells (AML), Salman et al. [58] evaluated anti-CD4 CAR NK cells in a NSG mice injected with luciferase-expressing MOLM-13 leukemic cells. These anti-CD4 CAR NKs showed 98% tumor regression by day 9, which was much more efficient as compared to unmodified NK treated mice [58].

Chen et al. [26] adapted a similar approach by targeting CD5, a marker expressed at the surface of a majority of T-cell malignancies including T-ALL and T cell lymphomas [100,101]. Anti-CD5 CAR NK cells inhibited and controlled cancer progression in xenograft mouse models of T-ALL but failed though to eradicate established tumor cells [26]. Moreover, the CAR NK cells did not persist since they were not detected 30 days post-injections, again emphasizing that CAR NK cell expression is transient. Alternatively, anti-CD5 CAR T and NK cells as reported by Mamonkin et al. [27] also allowed inhibition of disease progression in a T-ALL xenograft model. Maciocia et al. [102] choose another strategy to avoid CAR T cell-induced immunodeficiency in T cell lymphomas when targeting malignant T cells. Since TCR-αβ is highly expressed on T cell cancers [103], they suggested to target CAR T cells to one of two existing TCR β chain constant regions either encoded by TRBC1 or TRBC2 in a mutually exclusive manner [104,105]. Hence, T cell lymphoma cells which are normally monoclonal, will express either TRBC1 or TRBC2. They decided to target CAR T cells to TRBC1 in a model where the malignant T cells are homogenously expressing TRBC1. NSG mice injected with TRBC1^+^ Jurkat cells and TRBC2^+^ JKO cells, when treated with a TRBC1- directed CAR T cells, showed complete elimination of the Jurkat cells while the JKO T cells were the only surviving T cells. Moreover, co-injection of human PBMCs with TRBC1^+^ Jurkat cells in NSG followed by TRBC1 CAR T cell injection resulted in human non-CAR T cell survival confirming the persistence of healthy T cells during Jurkat elimination [102]. Since only one third of the healthy T cells expresses TRBC1, elimination of those healthy T cells in the clinic would not result in severe immunosuppression.

Normally the target cell for CAR T cell expression are mature T or NK cells. To allow persistence of the CAR T cells some authors introduced the CAR already at the level of the hematopoietic stem cell [106] since this results in continuous output of CAR-modified T cells and a long-term persistence of anti-cancer immunity. Larson et al. [107] introduced an anti-CD19 CAR into HSCs. The CAR-modified HSCs were transplanted into newborn NSG mice, which allowed to detect output in vivo of anti-CD19-CAR T cells in the blood of these humanized mice. Moreover, subsequent injection of malignant Raji B cells showed that the NSG mice engrafted with anti-CD19 CAR-transduced HSCs did not develop tumors even after 120 days in contrast to non-transduced HSC recipient mice, which developed huge-sized tumors and did not survive more than 60 days [107].

### 3.4. Avoiding Immune Response Against CAR T Cells in Vivo

Importantly, the early CARs harbor a murine antigen domain, which can potentially induce an immune response in the patients leading to premature elimination of the CAR T cells and might result in tumor relapse [8,108,109]. To overcome this issue, one team developed a fully human CD19-specific CAR, which proved functional for eliminating a human lymphoma xenograft in NSG mice [110]. Alabanza et al. [111] improved this design by humanizing the anti-CD19 CAR even more through insertion of a hinge and transmembrane domains (TM) of human CD8. This TM caused weaker T cell activation and lower cytokine release than the CD28 TM domain in a xenograft mouse model [111]. A recent clinical trial using this optimized CAR confirmed these results and concluded that the hinge and TM domain included in the CAR dictated the levels of cytokines released by the CAR T cells [112].

Blum et al. developed a humanized CAR against the B cell maturation Antigen (BCMA) [113]. BCMA-CAR T cells eradicated the tumor cells both in a multiple myeloma and a B cell lymphoma xenograft model [113]. BCMA CAR T cells were also able to target other B cells malignancies in humanized NSG mice in another study [114]. It is clear that a fully humanized CAR may reduce immune rejection compared to a murine-based CAR. In an attempt to reduce immune response even further, Lam and colleagues developed an anti-BCMA CAR carrying only a fully human heavy-chain variable domain instead of a complete scFv [115]. NSG mice were transplanted with MM.1S multiple myeloma cell line or with a human myeloma cell line. After establishing solid tumors, injection of anti-BCMA CAR T cells confirmed complete elimination of the tumors. Interestingly, long-term persistence and higher expansion of CAR T cells was only found in vivo when the CAR including only the heavy-chain of anti-BCMA scFv was combined with the 4-1BB co-stimulatory domain but not when including a CD28 co-stimulatory domain [115]. The same strategy was adapted for an anti-CD33 CAR for treatment of acute myeloid leukemia (AML) cells, which express high levels of the CD33 antigen [116].

CAR T cell therapy encounters in vivo multiple obstacles such as inhibitory signals from the tumor and its microenvironment. The latter can express the inhibitory ligands, programmed death ligand 1 (PDL-1) and 2 (PDL-2) for PD-1, which can is upregulated on activated CAR T cells [117] or NK cells. This PDL-1/PDL-2 binding to PD-1 h dampens the function and reduces persistence of these gene-modified cells [118,119,120,121,122,123,124]. To overcome this particular problem, CAR T and NK cells have been developed, in which inhibitory receptors were removed [125,126,127,128,129,130] or that express costimulatory signals or secrete factor that can re-activate the immune system such as inhibitors or cytokines [131,132]. One of those immune stimulating cytokines is IL-12P70, which was reported to increase CAR T cell activity [133,134,135]. Sachdeva et al. [136] achieved using an elegant strategy two objectives at once by gene editing of CAR T cells, in which they placed the IL-12P70 expression into the PDCD1 locus coding for PD1. By this means the secretion of IL-12P70 is under the control of PDCD1 regulatory elements, thus will only be expressed when the CAR T cells encounters the tumor antigen. Moreover, this concomitantly led to abolishment of PD1 expression on the CAR T cells, one major checkpoint of T cell function. In NSG mice xenografted with luciferase^+^ Raji cells, these authors demonstrated that the IL-12 secreting CAR T cells Knock-out (KO) for PDCD1 increased significantly antitumor activity and CAR T cell accumulation compared to CAR T cells KO for PDCD1 alone or CAR T cell counterparts [136]. These results might be explained by the controlled IL-12P70 secretion [133,134,135,137].

### 3.5. CAR T Cell Combination Therapy Evaluation

Very recently Parihar et al. [138] reported an interesting combinatorial approach to improve CAR T cell activity against solid tumors. They decided to combine CAR NK and CAR T cell therapy. NK cells strongly express NKG2D [139], a cytotoxicity receptor, for which the ligand is overexpressed on several solid tumors and on tumor-infiltrating myeloid-derived suppressor cells (MDSC) [140]. Binding of the ligand to NKG2D receptor reduced NK anti-tumor activity. The authors [138] showed that NK cells expressing a CAR against the NKG2D receptor [141], are able to eliminate the suppressive myeloid cells in the tumor and counteract in this way the immunosuppression to allow tumor-specific CAR T cells to persist and function in the tumor micro-environment. They used a xenograft model of neuroblastoma, in which they reconstituted a tumor micro-environment by co-injection of LAN-1 tumor cells with human MDSC cells subcutaneously into NSG mice. These were then treated with NKG2D directed NKs followed by GD2 (target on neuroblastoma) directed CAR T cells. In vivo MDSC cells were eliminated by the CAR NK cells from the tumor and increased recruitment of the CAR T cells into the solid tumors was demonstrated with strong tumor regression as compared to CAR T cell infusion alone. These data might argue for a combination of immune therapies for solid tumors. This is only one of the examples for combinatory approaches with CAR T cell therapy but multiple were tested in NSG xenograft models [142,143,144].

### 3.6. Mechanism of CAR T Cell Action

A major barrier to efficacy in CAR T cell therapy is T cell exhaustion, characterized by expression of inhibitory receptors and transcriptional and epigenetic alterations [145,146,147]. But the mechanism underlying CAR T cell exhaustion and dysfunction were up to recently not clear. Therefore, Lynn et al. [148] investigated this important issue using a tonically signaling CAR, driving healthy CAR T cells to exhaustion [12]. In these exhausted T cells dysregulation of Activator protein 1 (AP-1) transcription factor-binding motifs and increased expression of basic leucine zipper (bZIP) and interferon regulating factor (IRF) were detected. Importantly, these genes are implicated in the regulation of gene signatures of exhaustion. Lynn et al. [148] hypothesized exhaustion may be due to deficiency in c-jun/c-Fos/AP-1 heterodimers. Remarkably, c-jun overexpressing CAR T cell became resistant to exhaustion and upon antigen encounter, they showed higher production of IL-2 and IFNγ and increased levels of stem cell memory and central memory phenotype, characteristics of long-term persisting T cells. Moreover, using the Nalm6-GD2^+^ leukemia xenograft NSG model, c-jun overexpressing CAR T cells were superior for anti-tumor activity even when antigen expression on cancer cells was low. Importantly, c-jun-expressing CAR T cells demonstrated enhanced anti-tumor function in solid tumors. For example, c-jun^+^ Her2 targeted CAR T cells showed improved survival for 143B osteosarcoma tumor growth in vivo and strong in vivo expansion of these CAR T cells compared to control counterpart Her2 CAR T cells. Moreover, single cell analysis of infiltrating c-jun^+^ Her2 CAR T cells showed their strong proliferation, activation and downregulation of exhaustion markers. In conclusion, overexpression of c-Jun in CAR T cells avoided phenotypic and functional T cell exhaustion and accordingly increased anti-tumor control in several preclinical xenograft mouse models, which encourages clinical testing of Jun^+^ CAR T cells in the future. However, safety testing (off-target effects) is still required before entry into the clinical can be considered.

Xenograft NSG-based tumor models also aided in revealing why CAR T cell therapy induced tumor relapse in the clinic through tumor antigen loss or reduced expression of the CAR targeted antigen [112,149,150,151,152,153]. Hamieh et al. [153] used a NALM6 B cell acute lymphoblastic leukemia xenograft model (ALL), in which they infused a low dose of anti-CD19 CAR T cells leading to tumor relapse. In vivo CD19 expression was strongly reduced on the NALM6 cells, while surprisingly a fraction of the CAR T cells stained positive for CD19. These authors revealed that this was due to an active process in which the target antigen (CD19) is transferred from B cells to T cells, a mechanism called trogocytosis [153]. This diminishes antigen density on the cancer cells and thus their killing but additionally also leads to CD19^+^ T cell killing and exhaustion. This finding dictates the rationale for combinatorial targeting CAR T cell strategies.

## 4. In Vivo CAR T Cell Generation Using Lentiviral Vectors Targeted to Specific Human T Cells

### 4.1. Advantages of in Vivo CAR T Cell Generation

The delivery of therapeutic or relevant genes directly into the organism is called in vivo gene delivery. In vivo targeted CAR delivery to T cells would represent a big step forward in the field of cancer therapy.

Importantly, CAR transfer in vivo must be specific for the target T cell to avoid transfer in malignant cells and the risk of transducing antigen presenting cells (APCs), which might elicit a transgene specific immune response leading to elimination of CAR T cells. Freshly isolated T lymphocytes, though, are not susceptible to classical VSV-G pseudotyped lentiviral vector transduction, unless they are stimulated through the TCR to allow efficient gene transfer [50,154]. This ex vivo transduction and amplification process to generate CAR T cells, clearly changes their phenotype and long-term in vivo persistence before infusion. Moreover, ex vivo CAR T cell therapy remains a personalized treatment since it requires ex vivo production of gene-modified autologous cells using high doses of vectors. These manufacturing processes are extremely costly. In vivo administration of the vector would omit this labor-intensive and costly ex vivo process. In summary, in vivo gene therapy, consisting of a single injection of a vector encoding CARs into the blood stream, might make CAR T cell therapy more broadly available to patients.

### 4.2. Evaluation of in Vivo CAR T Cell Generation in Humanized Mice

Humanized mice provide a unique system to evaluate the genetic modification by vectors targeted to specific cell types in vivo. As stated already above, the immunodeficient NOD/SCID, γc^−/−^ (NSG) mice [155,156] allow high level engraftment of human HSCs and reconstitution with human lymphoid immune cells but are still refractory in the human myeloid lineage [157]. Meanwhile, improved humanized mouse models have been reported, which supported much better myeloid differentiation [158,159]. In the future, these will be the better models to evaluate specificity of targeted vectors for transduction of subtypes of T cells upon in vivo administration. But for the moment study of in vivo CAR T cell engineering, relied on the well characterized NSG mouse model.

As mentioned above it would be of great benefit if the CAR-encoding vectors could be injected directly in vivo to transduce the cells of choice, e.g., human CD4^+^ or CD8^+^ T cells. LVs were retargeted specifically to human CD4 and CD8 T cells through introduction of a scFv or a Designed Ankyrin repeat protein (DARPIN) directed against CD4 or CD8 epitopes into the measles virus (MV) glycoprotein H. These CD4-MV and CD8-MV retargeted vectors showed, exclusive gene transfer into the CD4^+^ or CD8^+^ subset of hT cells, respectively, in vitro in human PBMCs. Remarkably, also in vivo upon systemic delivery in NSG mice humanized with hPBMCs targeted gene transfer into the CD4^+^ or CD8^+^ T cells was confirmed [160,161]. Additionally, CD4-MV LVs also specifically targeted CD4 T cells in HSC-humanized NSG mice [161].

The same research team developed LVs pseudotyped with receptor-retargeted Nipah virus glycoproteins (NiV-LVs) [162]. These NiV-LVs could be produced at higher titers and were not inactivated in vivo since in the human serum no neutralizing antibodies against Niv are present.

**Figure 5:**
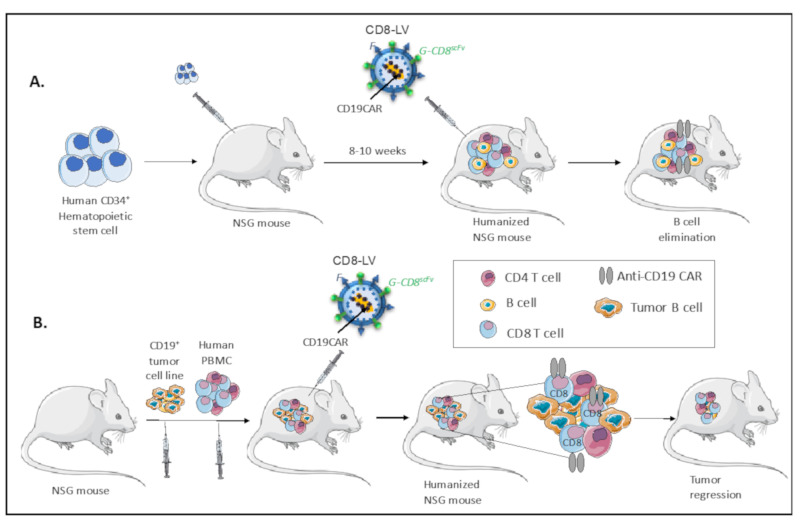
In vivo CAR T cell generation in humanized mice. (**A**) Cord blood CD34^+^ cells were injected into NSG mice and humanized for 8-10 weeks before injection of the CD8-targeted (CD8NiV)-LV encoding for a CAR-directed against CD19 present on B cells. Mice were sacrificed at 5-12 weeks for FACS analysis of the immune cells. The CAR expression was revealed exclusively in hCD8 T cells, which were amplified in vivo through contact with CD19^+^ B cells. Upon contact these gene-modified CD8 T cells were able to eliminate the human B cells in the different hematopoietic tissues (blood, spleen, bone marrow). (**B**) B cell line xenografted NSG mice, were subsequently infused with human PBMCs. Upon tumor formation and human immune cell reconstitution, the NSG mice were injected with CD8-targeted (CD8NiV)-LV encoding for a CAR-directed against CD19 present on tumor B cells. The CAR expression was revealed exclusively in hCD8 T cells, which were amplified in vivo through contact with CD19^+^ on the tumor B cells. Upon contact these gene-modified CD8 T cells were able to eliminate the tumor B cells.

Importantly, Pfeifer et al. has very recently performed a first step toward in vivo reprogramming of CAR T cells using CD8 T cell directed NiV-LVs encoding for an anti-CD19 CAR. A single administration of the anti-CD19 CAR encoding CD8NiV-LVs in the blood stream of HSC-humanized NSG mice generated anti-CD19 CAR-expressing CD8 T cells in vivo, which induced the elimination of the CD19^+^ B cells from all hematopoietic tissues (Figure 5A; [163]). The generation of these CAR T cells in vivo was associated with the induction of CRS in some mice, similar to patients infused with CAR T cells, which underlines the need for supplemental optimization [163].

More recently, this team evaluated the same CD8NiV-LV delivering the anti-CD19 CAR in an NSG mice engrafted with CD19^+^ Nalm-6 tumor cells, followed by injection with human PBMCs [164]. A single injection of this CD8 targeted LV was sufficient to eliminate CD19^+^ Nalm-6 tumor cells, whereas in control animals tumor cells expanded in a uncontrolled manner [164]. Surprisingly, they detected also anti-CD19 CAR expression at the surface of NKT cells in vivo, since these cells also express the CD8α chain, the target of the CD8NiV-LV particles. This study provides for the first time a clear evidence of in vivo anti-CD19 CAR T cell generation in a cancer xenograft model (Figure 5B).

Although the humanized mice in this context is extremely useful as a preclinical model for in vivo evaluation of vectors targeted to specific immune cells, we have to be prudent in directly translating these results to patients since these mice lack a fully functional human immune system. Clearly, further testing in immune competent model (e.g., non-human primates) is warranted before moving to a clinical trial with in vivo CAR T cell gene therapy [165].

## 5. Conclusions

Humanized mice have been instrumental in evaluating safety, efficacy, and specificity of CAR T and NK cell therapy directed against various cancer antigens on numerous cancers. What’s more, they often provide the missing link between the proof of concept of innovative strategies to overcome current limitations in CAR T cells and their translation into the clinic. For the moment, CAR T cells have been approved in the clinic for some hematological malignancies, and ongoing trials hopefully will extend CAR T application to solid tumors, for which new treatment options are urgently needed. Importantly, NK CAR T cells are not approved as a clinical drug yet and preclinical and clinical testing is still required before they will be available to patients. Among these hurdles, CAR T and NK cells encounter immune responses, inhibitory signals from the tumor cells and tumor microenvironment, toxic side-effects, and loss of long-term persistence among others. The field is actively looking for solutions to these obstacles by multiple inventive approaches, including gene editing techniques and in vivo generation of CAR T cells to improve accessibility of the CAR T cell therapy to more patients. In the future, improved mice models that mimic even closer human hematopoiesis and immune response [166] will help the field to address questions otherwise still unanswered.
